# Corrosion Behavior of Zn, Fe and Fe-Zn Powder Materials Prepared via Uniaxial Compression

**DOI:** 10.3390/ma14174983

**Published:** 2021-08-31

**Authors:** Radka Gorejová, Ivana Šišoláková, Pavol Cipa, Róbert Džunda, Tibor Sopčák, Andrej Oriňak, Renáta Oriňaková

**Affiliations:** 1 Department of Physical Chemistry, P. J. Šafárik University in Košice, Moyzesova 11, 040 01 Košice, Slovakia; radka.gorejova@student.upjs.sk (R.G.); pavol.cipa@student.upjs.sk (P.C.); andrej.orinak@upjs.sk (A.O.); renata.orinakova@upjs.sk (R.O.); 2 Institute of Material Research, Slovak Academy of Science, Watsonova 47, 040 01 Košice, Slovakia; rdzunda@saske.sk (R.D.); tsopcak@saske.sk (T.S.)

**Keywords:** iron, zinc, metallic powders, biodegradation, corrosion

## Abstract

Powder metallurgy is one of the most prevalent ways for metallic degradable materials preparation. Knowledge of the properties of initial powders used during this procedure is therefore of great importance. Two different metals, iron and zinc, were selected and studied in this paper due to their promising properties in the field of biodegradable implants. Raw powders were studied using scanning electron microscopy (SEM) coupled with energy dispersive spectrometry (EDX). Powders (Fe, Zn and Fe-Zn in a weight ratio of 1:1) were then compressed at the pressure of 545 MPa to the form of pellets with a diameter of 1.7 cm. Surface morphology and degradation behavior in the Hanks´ solution were studied and evaluated. Electrochemical polarization tests along with the static immersion tests carried out for 21 days were employed for corrosion behavior characterization. The highest corrosion rate was observed for pure Zn powder followed by the Fe-Zn and Fe, respectively. A mixed Fe-Zn sample showed similar properties as pure zinc with no signs of iron degradation after 21 days due to the effect of galvanic protection secured by the zinc acting as a sacrificial anode.

## 1. Introduction

Biomaterials can be described as widely used materials in current medical practice for the treatment and replacement of those tissues and organs that have been damaged or undergone degeneration [1,2]. The use of biomaterial has affected humans for thousands of years. Even though the first biomaterials used in the field of medical therapeutics date back to over 32,000 years, most of the biomaterial applications have occurred over the past 2000 years [3]. Currently, a wide range of biomaterials is used for disease and injuries treatment. The most commonly used biomaterials devices in medicine are various types of implants such as dental implants, vascular stents, synthetic heart valves, and medical devices such as biosensors, cardio stimulators, etc. [4,5].

The most important factor that distinguishes a biomaterial from other materials is its ability to exist in contact with tissues without causing an unacceptable change in the body [6]. This feature of the material is called biocompatibility. The definition of biocompatibility reflects that the used material does not have to be toxic, allergenic, carcinogenic and mutagenic [7]. Currently, bio-inert materials such as stainless steel, titanium and cobalt-chromium alloys are commonly used in orthopedic surgery [8,9,10]. These inert materials do not initiate a host response in biological tissue. Recently, biodegradable materials represent the unique field on which physicists, chemists, material engineers and medical communities are intensively focused [2,11,12]. Biodegradable materials can overcome the shortcomings associated with temporary implants, such as post-operational inflammation, thrombus formation and additional operation to remove implants with a transient function [13]. Another advantage of biodegradable materials is the possibility to develop material with optimal degradation time leading to degradation and replacement by host tissue over a given time. The most commonly used biodegradable materials include polymers, metals and ceramics [2,14,15]. The advantage of metals and their alloys in comparison to polymers or ceramics is in their higher strength and toughness [16].

Magnesium alloys represent a large class of biodegradable materials with a fast degradation rate under the physiological condition. Various studies were focused on the surface modification of these alloys including phosphating treatment, electrodeposition and polymer coating to slow down the corrosion rate [17]. Using the various mentioned modifications, it is possible to control the degradation rate of magnesium alloys depending on the type of implant that makes these alloys suitable candidates for the implant material. Iron is an essential element for necessary biological functions, mainly for the transfer of oxygen in human blood. As a conventional metal, iron exhibits better mechanical performance than most polymer materials and other metals with no local or systemic toxicity [12]. However, a faster corrosion rate is required for corrodible iron implants. Recently, valuable efforts have been made to affect corrosion rate by alloying or surface modifications of pure iron [18,19]. Herewith, the corrosion rate of iron can be increased to the required values [20]. Zinc supports the immune system, and it is a component of many food supplements; therefore, it is considered a non-toxic element. The recommended daily dose of zinc is about 40 mg, but short-term values of up to 100 mg do not cause significant health problems. Therefore, zinc can be considered a suitable biodegradable implant material because of its good biocompatibility [21]. It is also well-known that the corrosion rate of zinc is faster than the iron corrosion rate. Therefore, by modification of Zn and Fe powders or by creating their mixture, a biomaterial with the required corrosion properties can be prepared. Several papers studied iron composite materials or alloys with the addition of manganese, tungsten, palladium, silver or carbon nanotubes (CNT), for example [22,23,24]. However, only several papers deal with the absorbable Fe-Zn materials [25,26,27,28] even though both these elements are biocompatible and have a great potential in the field of biodegradable metals.

Another important property of biodegradable implants which is currently intensively studied is porosity. The biodegradable porous metals provide unprecedented opportunities for fulfilling the requirement for a suitable bone-implant [29]. Porous implants can perfectly replace bones because of their structure and mechanical properties leading to required tissue growth [30,31].

In this paper, the corrosion properties of Fe-Zn powder were examined and compared with the corrosion properties of pure Fe and Zn powders. The morphology of Zn, Fe and Fe-Zn powders and pellets was studied via scanning electron microscopy (SEM) coupled with the energy dispersive analysis (EDX). The corrosion behavior of the samples prepared by uniaxial compression was therefore determined using electrochemical and static immersion degradation tests. This study aims to prepare and characterize the material consisting of Fe-Zn with an optimal corrosion rate for biodegradable implants preparation and to understand the degradation process ongoing on the surface of the mixed Fe-Zn compressed sample. The ability to design the implant with a desirable corrosion rate by adjusting weight ratios of initial powders in the mixture can therefore lead to the preparation of a tailor-made biodegradable implant.

## 2. Materials and Methods

### 2.1. Fe, Zn and Fe-Zn Pellet Preparation

Metallic cylindrical pellets with a diameter of Ø = 1.7 cm and a height of 0.4 cm were prepared via uniaxial compression from raw powders. Samples made from pure Fe (99.50% purity, Alfa Aesar, Haverhill, MA, USA), pure Zn (99.99% purity, Centralchem, Bratislava, Slovakia) and their mixture in a weight ratio of 1:1 (Fe-Zn; mechanically mixed for 10 min) were compressed using a hydraulic press (Redats H-380, P.H.U Szczepan, Krakow, Poland) at 545 MPa.

### 2.2. Surface Morphology, X-ray and EDX Analysis

Macroscopic images were obtained using optical microscopy (Dino-Lite Premier AM4013MT, Dino-Lite AM4815ZT and Dino-Lite AM4515T8, ~20–900× magnification, 1.3 MPx, Dino-Lite, Delmenhorst Netherlands). The scanning electron microscope (SEM) coupled with an energy dispersive spectrometer (EDX) (JEOL JSM-7000F, Tokyo, Japan with EDX INCA and Tescan VEGA3, Brno, Czech Republic) was used to study the surface morphology and surface chemical composition of pure Fe, pure Zn and Fe-Zn initial powders and compressed samples. Powder particle size distribution analysis was performed using ImageJ software. Phase distribution was studied by X-ray diffraction (XRD) using diffractometer PhilipsX’ PertPro (Cu Kα radiation, 40 kV, 50 mA, 2θ between 10 and 90°, Philips, The Netherlands).

### 2.3. Corrosion Measurements

#### 2.3.1. Electrochemical Tests

To determine the corrosion rate of the prepared samples, potentiodynamic polarization tests were carried out in Hanks´ solution (8 g·L^−1^ NaCl, 0.4 g·L^−1^ KCl, 0.14 g·L^−1^ CaCl_2_, 0.06 g·L^−1^, MgSO_4_·7H_2_O, 0.06 g·L^−1^ NaH_2_PO_4_·2H_2_O, 0.35 g·L^−1^ NaHCO_3_, 1.00 g·L^−1^ Glucose, 0.60 g·L^−1^ KH_2_PO_4_ and 0.10 g·L^−1^ MgCl_2_·6H_2_O) at 37 ± 2 °C and pH = 7.4 ± 0.2. An argentochloride electrode (Ag/AgCl/KCl (3 mol·L^−1^)) was used as a reference electrode, a platinum as a counter electrode and the samples as a working electrode. All samples were ultrasonically cleaned in acetone and ethanol for 10 min each before measurements and examined in triplicate. Multichannel potentiostat Autolab M204 (Metrohm, Herisau, Switzerland) was used, and measurements were conducted at a 0.1 mV·s^−1^ scan rate. Open circuit potential (OCP) was measured for 60 min before the corrosion measurement to ensure the potential stability of the studied system. The corrosion rate was calculated using Equation (1)

(1) $$CR=\frac{j_{corr\times EW\times K\;}}{\rho }$$

where *CR* stands for corrosion rate (mmpy); *j_corr_* is corrosion current density (μAcm^−2^). Since the presented curves do not meet the prerequisites for the Tafel fitting, data were calculated using a non-Tafel evaluation from the cathodic branch of the polarization curve. *EW* is the equivalent weight (32.69 g·eq^−1^ for Zn; 27.92 g·eq^−1^ for Fe and 30.31 g·eq^−1^ for Fe-Zn determined based on the weight percentage ratio of each element in the mixed sample); *ρ* is the sample density and *K* is a constant (3.27 × 10^−3^) determining CR units.

#### 2.3.2. PH and Ions Concentration Determination

All samples were ultrasonically cleaned in acetone and ethanol for 10 min, oven-dried at 55 °C and weighed. Static immersion tests were carried out for 21 days at 37 ± 2 °C. A total of 50 mL of Hanks´ solution [32] with uniform access to the whole sample surface was used as a corrosive medium and was replaced every 7 days. Samples were removed from testing corrosive after the solution, rinsed with distilled water, ultrasonically cleaned in ethanol for 10 min and returned to the fresh medium. After 7 and 21 days, the pH of the medium was measured, and the surface morphology of the corroded samples was studied. Ions’ concentration in corrosive media was evaluated using the atomic absorption spectroscopy method (AAnalyst 100, Perkin Elmer, Waltham, MA, USA).

## 3. Results

### 3.1. Fe, Zn and Fe-Zn Powders and Compressed Samples Characterization

**Figure 1:**
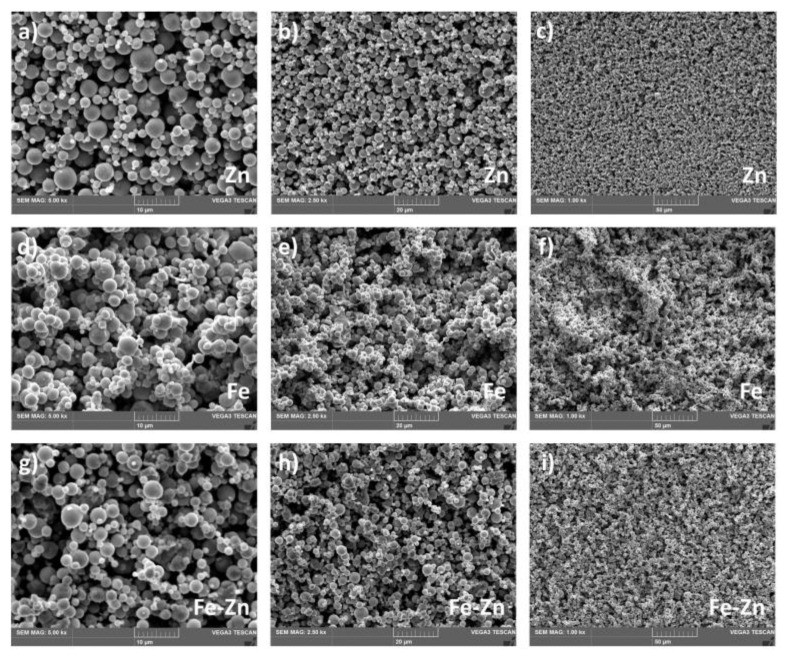
SEM micrographs of Zn (**a**–**c**), Fe (**d**–**f**) and Fe-Zn (**g**–**i**) powders at 5000×, 2500× and 1000× magnification.

**Figure 2:**
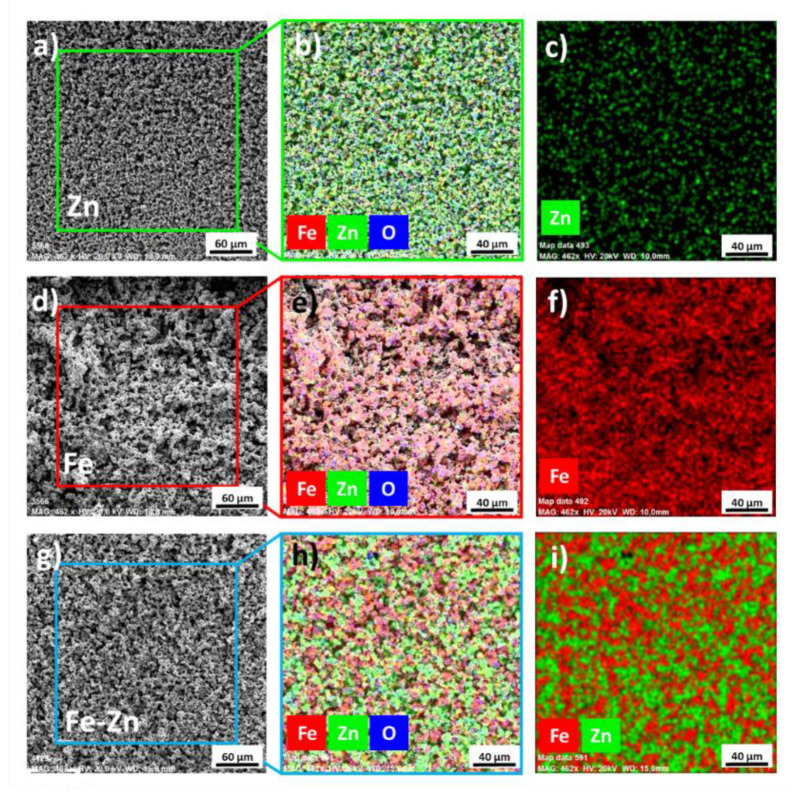
Chemical composition and particle distribution of Zn (**a**–**c**), Fe (**d**–**f**) and Fe-Zn (**g**–**i**) powders obtained from EDX analysis.

The morphology of Fe, Zn and Fe-Zn powders along with the surface morphology of the compressed samples was studied using scanning electron microscopy (SEM) (Figure 1). EDX analysis was employed to study the powder particles distribution further (Figure 2).

Zinc powders consisted of homogenously distributed spherical particles with an average diameter of 3 ± 2 µm (Figure 1a–c). Very low to no signs of particle aggregation were observed. On the other hand, iron particles tend to create aggregates composed of smaller powder particles due to their magnetic properties (Figure 1d–f). The average Fe powder particle diameter size was 2.4 ± 1.5 µm. In the Fe-Zn powder mixture, particles were well homogenized (Figure 2i). Besides the metals, oxygen was also detected by the EDX analysis (Figure 2b,e,h). The average particle size in the Fe-Zn mixture was 2.8 ± 1.7 µm.

**Figure 3:**
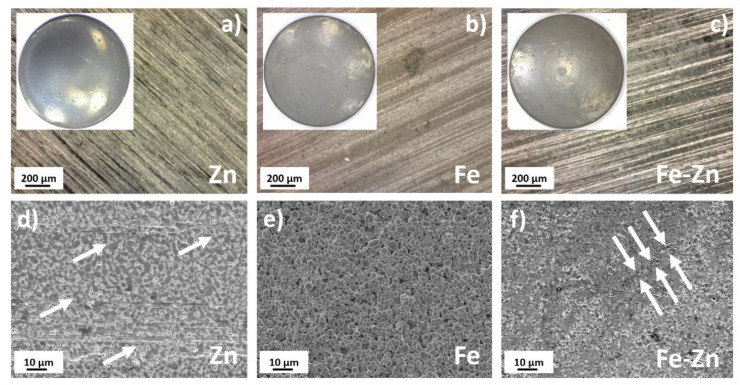
Macroscopic optical photographs of Zn (**a**), Fe (**b**) and Fe-Zn (**c**) samples with the corresponding micrographs showing the material surface in detail (**d**–**f**). White arrows point at the local defects and cracks that appeared after compression.

Initial powders described above were subsequently compressed into pellets with a diameter of 1.7 cm and studied. Optical photographs along with the SEM micrographs of the material surface after compression are depicted in Figure 3. Horizontal lines visible on the sample´s surfaces were created during the compression process. The height of the compressed pellets was ~0.4 mm and did not significantly differ for Fe, Zn or Fe-Zn specimens. The compaction of Zn powder led to the formation of the homogenous, rather uniform surface morphology with no precisely defined grain boundaries (Figure 3d). Only solitary pores in a microscopic range were present (highlighted with white arrows). Spherical powder particles with clearly recognizable grain boundaries and gaps between grains were present in the case of iron (Figure 3e). The surface of the sample compressed from the Fe-Zn mixture (Figure 3f) was more reminiscent of the Zn sample; however, bigger iron grains appeared on the surface. The level of porosity was higher than that of a pure Zn sample with microscopic pores evenly distributed through the whole surface. When several micropores combined, longitudinal cracks appeared (Figure 3f, white arrows). These defects may play an important role in the material degradation behavior, and it is known that their influence is also important besides the material composition and design [33]. Their development after further heat treatment, which must be done before use in vivo, should be therefore thoroughly studied.

**Figure 4:**
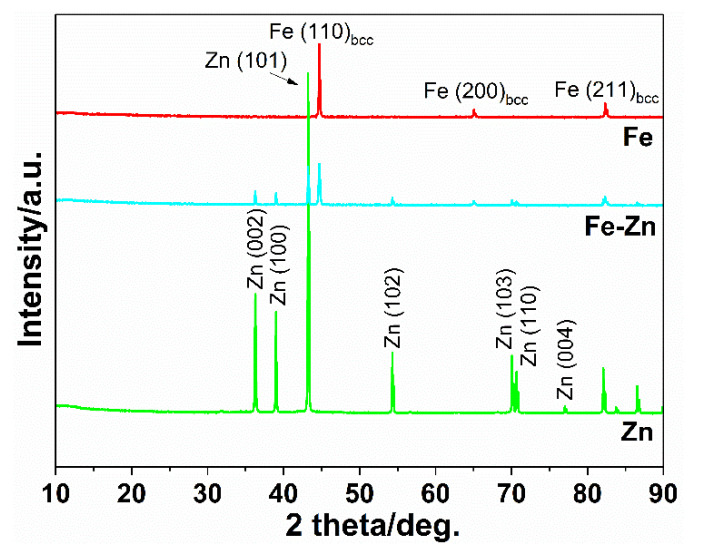
XRD spectrum of Zn, Fe and Fe-Zn compressed metallic powders.

Peaks for Zn and Fe were obtained (Figure 4). In the mixed Fe-Zn sample, both peaks for Zn and Fe were present with lowered intensity when compared to the samples made of pure metals. No signs of local impurities were detected which can emerge during the sample preparation process; only the peaks for pure metals were present.

### 3.2. Degradation Behavior of Compressed Fe, Zn and Fe-Zn Powders

**Figure 5:**
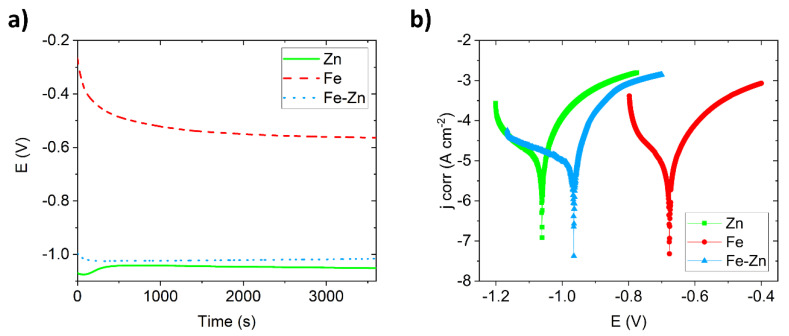
Determination of the open circuit potential of Zn, Fe and Fe-Zn compressed pellets in Hanks´ solution at 37 ± 2 °C was measured for 1 h (**a**). Potentiodynamic polarization curves for Zn, Fe and Fe-Zn compressed pellets measured at the same conditions (**b**).

Degradation in Hanks´ solution simulating body fluids were studied electrochemically. Open circuit potential (OCP) was stabilized over 1 h to reach the equilibrium state before further corrosion measurement (Figure 5a). Each sample reached a stable state even before one hour. Zn and Fe-Zn samples were stabilized after ~500 s while the sample made of pure iron after ~1000 s. The most positive potential was observed for the Fe sample starting around −0.300 V which subsequently decreased to the lower value (−0.564 V). The starting potential of both Zn and Fe-Zn samples was under −1.0 V. While in the case of pure zinc this value slightly increased in the initial period, in the case of Fe-Zn, it was the other way around. After one hour, the most negative values of potential were observed for Zn, followed by the Fe-Zn and Fe, respectively.

Potentiodynamic polarization curves obtained in Hanks´ solution at 37 ± 2 °C are depicted in Figure 5b. The lowest corrosion potential (*E_corr_*) was observed for the Zn sample, followed by the Fe-Zn and Fe samples, respectively. Corrosion characteristics (corrosion potential *E_corr_*, corrosion current density *j_corr_*, corrosion rates and polarization resistance) are summarized in Table 1. The highest corrosion current density was measured for the Fe-Zn sample (43 ± 2.9 µA·cm^−^²), which was close to that of pure zinc (37 ± 2.9 µA·cm^−^²), while corrosion current density for the Fe sample was the lowest. Similarly, corrosion potentials of pure zinc and Fe-Zn were shifted to the more negative potentials; however, the most negative value was observed for zinc. Corrosion rates were evaluated using the non-Tafel evaluation and represent approximate values since the polarization curves did not meet the criteria for Tafel evaluation [34]. The highest degradation speed was observed for pure zinc followed by the Fe-Zn and Fe samples, respectively. This corresponds to the highest value of polarization resistance obtained for pure iron (339 ± 58 Ω). As in the case of corrosion potentials and corrosion current density, the corrosion rate of the Fe-Zn mixed sample got closer to that of pure zinc rather than pure iron.

**Figure 6:**
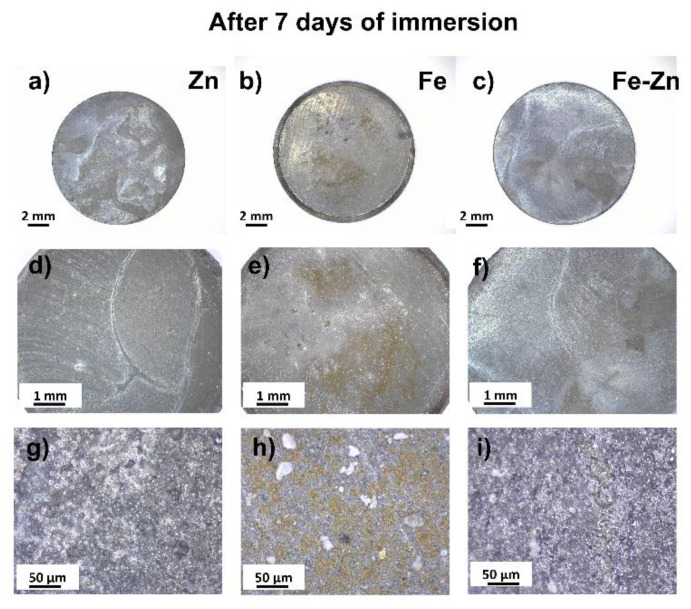
Optical photographs of corroded Zn (**a**,**d**,**g**), Fe (**b**,**e**,**h**) and Fe-Zn (**c**,**f**,**i**) samples after 7 days of immersion in simulated body fluids (Hanks´ solution at 37 ± 2 °C, pH = 7.4 ± 0.2).

To study the degradation behavior of prepared samples in vitro, long-term immersion tests were carried out for 21 days. The surface morphology of samples corroded for 7 days is depicted in Figure 6. White corrosion products in the form of spherical deposits (Figure 6g,i) were present in the case of Zn and Fe-Zn. Brown deposits appeared on the surface of the Fe sample locally (Figure 6e) with homogenously distributed white crystal-like deposits (Figure 6h). The mixed Fe-Zn sample behaved similarly to the pure zinc sample, and either orange or brown corrosion products were not present after 7 days of immersion.

**Figure 7:**
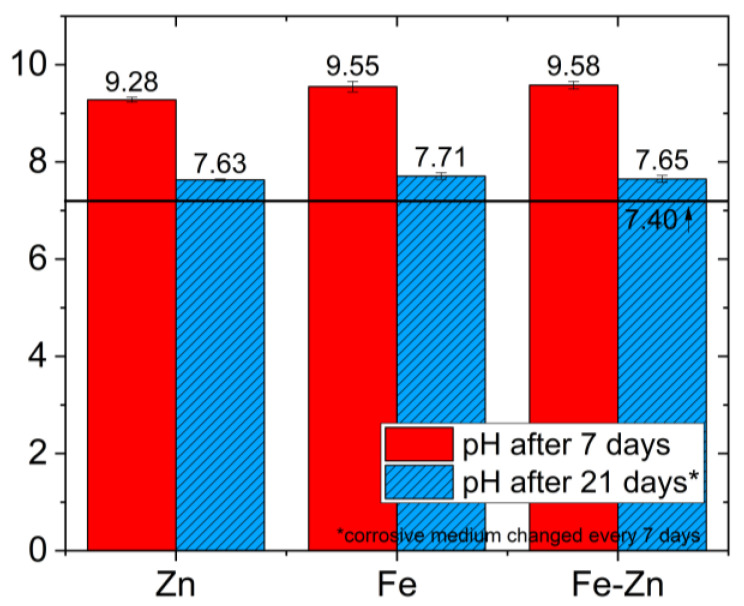
Comparison of changes in pH after 7 and 21 days of immersion in simulated body fluids for Zn, Fe and Fe-Zn compressed powders samples.

Changes in the pH of Hanks´ solutions were also determined and are summarized in Table 2. While the rapid increase in pH was observed in the first initial week (ΔpH_7 days_ = 1.88–2.18), rather mild growth appeared in the third week (ΔpH_21 days_ = 0.23–0.25). All of the samples overcome the value of 9 with the most prominent increase found in the Fe-Zn sample. The pH of the solution with the pure iron sample reached the highest value after 21 days (7.71 ± 0.07), while the pH of solutions with both Zn and Fe-Zn samples differed only slightly (Figure 7). Ions’ concentration in the solution was determined using the atomic absorption spectroscopy method, and the results after 7 and 21 days of immersion (with medium refreshed every 7 days) are summarized in Table 2. The values of iron in the mixed Fe-Zn sample after 7 and also 21 days of immersion were below the detection limit, while the values obtained for Zn were similar in the pure Zn and Fe-Zn samples (~6 mg·L^−1^).

**Figure 8:**
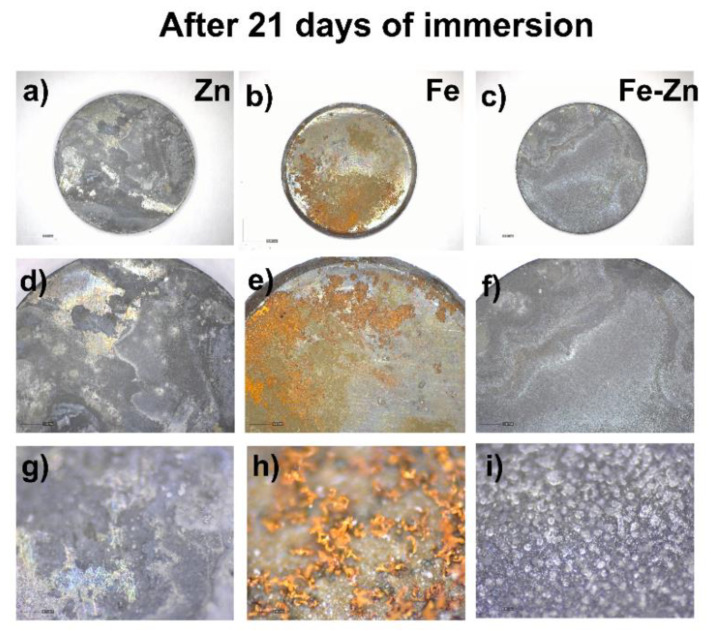
Optical photographs of corroded Zn (**a**,**d**,**g**), Fe (**b**,**e**,**h**) and Fe-Zn (**c**,**f**,**i**) samples after 21 days of immersion in simulated body fluids (Hanks´ solution at 37 ± 2 °C, pH = 7.4 ± 0.2).

The spread of corrosion after another two weeks is depicted in Figure 8. Degradation progressed in all studied samples, and the corrosion deposits covered the majority of the sample surface (Figure 8a–c). The same trend as after 7 days was observed where Zn and Fe-Zn remained covered in white deposits (Figure 8g,i), while corrosion of the Fe sample advanced, and more protruding products were formed and raised on the surface (Figure 8h).

## 4. Discussion

Three different types of metallic samples were prepared from raw powders, and their degradation properties were studied. Initial Fe, Zn and Fe-Zn powders were studied using SEM and EDX methods. A difference in the starting powders was detected. While the Fe powder tends to create aggregates due to its magnetic character, zinc powder particles were homogenously distributed without the signs of aggregation. Aggregation was suppressed in the Fe-Zn mixture due to the zinc presence. EDX analysis revealed that the oxygen corresponding to metals’ oxides is present even in the initial powders. Powders can be oxidized during manipulation and sample preparation in the air environment. Manipulation in the inert atmosphere, e.g., argon or nitrogen, can prevent this oxidation and should be further used. Pellets from these powders were hydraulically pressed at 545 MPa to obtain experimental samples in the form of pellets with a diameter of 1.7 cm. Compressed specimens did not differ in height, and only color differences can be visible when the surface of the pure Zn sample appeared lighter than others due to the nature of zinc. X-ray diffraction analysis confirmed the presence of both Fe and Zn in the mixed samples. Peaks for Fe(110)_bcc_, Fe(200)_bcc_ and Fe(211)_bcc_ were identified in the spectrum of pure Fe powder, which corresponds to the literature [35,36,37]. Zn (002), Zn(100), Zn(102), Zn(103), Zn(110) and Zn(004) peaks were identified in the spectrum of pure zinc with a hexagonal closed pack structure which is following the literature [38,39,40]. All of the peaks identified in either the Fe or Zn spectrum were also present in the spectrum of the Fe-Zn sample without substantial shift and with the suppressed intensity.

Corrosion properties of the studied samples were determined both electrochemically and by the in vitro immersion studies. Electrochemical polarization tests showed the difference in the corrosion rates between the Fe and Zn samples according to their chemical nature; however, we were mainly interested in the behavior of mixed Fe-Zn. Even though the mixture was prepared in the weight ratio of 1:1, corrosion properties of the mixed sample were shifted to those of pure zinc. The slowest degradation was observed for pure Fe followed by the Fe-Zn and Zn samples, respectively. The difference between the CR of pure Zn and the Fe-Zn samples was only mild. The shift of the OCP and potentiodynamic curves were similar for Zn and Fe-Zn samples also. The most visible difference was spotted in the initial stage of OCP measurement when the rapid decrease in potential was observed for Fe and Fe-Zn samples, while the increase was obtained in the Zn sample suggesting its passivation during the early stages of the measurement. The cathodic branch of the zinc polarization curve corresponds to the oxygen reduction reaction (Equation (3)), where the electrons produced during the anodic process (Equation (2)) are consumed and lead to the formation of corrosion products. The most shallow region in the cathodic part of the curve nearing the corrosion potential was found in the Fe-Zn sample. In the area of physiological pH, where the formation of oxides and hydroxides of Zn is expected thermodynamically, the formation of an effective passivation layer was not confirmed. In the anodic branch, the rapid increase in the current density was observed for all the studied specimens in the anodic branch which is related to the metal dissolution.

**Figure 9:**
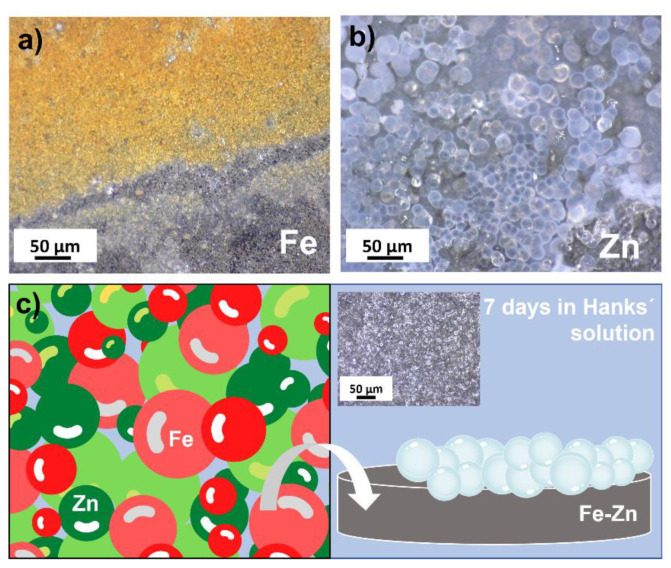
Corrosion products found on the sample made of pure iron (**a**), pure zinc (**b**) and schematic representation of corrosion process of the Fe-Zn sample (**c**).

Degradation rates could not be calculated from the immersion due to the continual increase in the weight of studied samples caused by the corrosion products’ formation. Longer immersion time, therefore, needs to be studied in the future to evaluate degradation via the mass loss experiments. Mechanical removal of corrosion products was not used in order not to harm the metallic surface before further degradation. Besides the corrosion rates, the character and appearance of the corrosion deposits indicated the same results as electrochemical tests. While on the surface of pure iron, brownish corrosion deposits were found (Figure 9a), spherical white deposits were found both on the surface of the pure zinc and Fe-Zn samples (Figure 9b). This observation was of great importance in terms of understanding the ongoing corrosion process in the Fe-Zn sample. These results indicate that during the corrosion of the mixed Fe-Zn sample, no iron oxidation occurs, and the results were supported by the AAS analysis of Hanks´ solution after 7 and 21 days. The values for iron ions detected in the mixed sample were below the detection limit, while these for zinc ions were similar in the case of the pure Zn and Fe-Zn samples. White spherical deposits created on the surface of studied samples are most likely calcium phosphates originating from the Hanks´ solution, which has been also reported earlier in the study of degradation in simulated body fluids [41].

No signs of iron degradation products on the surface of the Fe-Zn samples indicate the predominant reaction of zinc instead of iron in the Hanks´ solution. During the biodegradation of zinc in simulated body fluids, the following reactions take place [41,42,43]:

(2) $$2Zn\;\to 2Zn^{2+}+4e^{-}$$

(3) $$O_{2}+2H_{2}O+4e^{-}\;\to 4OH^{-}$$

(4) $$Zn^{2+}+2OH^{-}\to Zn{\left(OH\right)}_{2}$$

(5) $$Zn{\left(OH\right)}_{2}\to ZnO+H_{2}O$$

(6) $$6\;Zn{\left(OH\right)}_{2}+Zn^{2+}+2Cl^{-}\;\to 6Zn{\left(OH\right)}_{2}\cdot ZnCl_{2}$$

(7) $$4ZnO+4H_{2}O+Zn^{2+}+2Cl^{-}\;\to 4Zn{\left(OH\right)}_{2}\cdot ZnCl_{2}$$

(8) $$3Zn^{2+}+2HPO_{4}^{2-}+2OH^{-}+2H_{2}O\to \;Zn_{3}{\left(PO_{4}\right)}_{2}{\left(H_{2}O\right)}_{4}$$

(9) $$5Zn^{2+}+2HCO_{3}^{-}+8OH^{-}\to \;Zn_{5}{\left(CO_{3}\right)}_{2}{\left(OH\right)}_{6}+2H_{2}O$$

The reaction starts with the anodic dissolution of zinc to Zn^2+^ ions (Equation (2)). Electrons produced in this step are consumed during cathodic oxygen reduction (Equation (3)), and corrosion products (Zn(OH)_2_, ZnO) are formed (Equations (4) and (5)). In Cl^−^ rich solutions, such as Hanks´ solution, soluble chlorides are produced (Equations (6) and (7)) [41,44,45]. Besides the chlorides, phosphates and carbonates are created by the reaction of phosphate ions originating from Hanks´ solution and released Zn^2+^ cations (Equation (8)) [42]. Degradation of iron starts with anodic iron oxidation (Equation (10)) and cathodic reduction reaction (Equation (11)):

(10) $$Fe\;\to {\;Fe}^{2+}{+2e}^{-}$$

(11) $$O_{2}+2{\;H}_{2}O+4{\;e}^{-}\to 4{\;OH}^{-}$$

The next step is the creation of iron hydroxides (Equations (12)–(14)) and magnetite (Equation (15)):

(12) $${Fe}^{2+}+2{\;OH}^{-}\to Fe{\left(OH\right)}_{2}$$

(13) $${Fe}^{2+}\;\to {\;Fe}^{3+}+e^{-}$$

(14) $${Fe}^{3+}+3{\;OH}^{-}\to Fe{\left(OH\right)}_{3}$$

(15) $$Fe{\left(OH\right)}_{2}+2\;FeO\left(OH\right)\to {Fe}_{3}O_{4}+H_{2}O$$

Corrosion of iron in Hanks´ solution specifically was described by Zhang [46] (Equations (16)–(22)):

(16) $$Fe{\left(OH\right)}_{2}+{Cl}^{-}\to FeClOH+{OH}^{-}$$

(17) $$FeClOH+H^{+}\to {Fe}^{2+}+{Cl}^{-}+H_{2}O$$

(18) $$Fe{\left(OH\right)}_{3}+2{\;Cl}^{-}\to {FeCl}_{2}OH+2{\;OH}^{-}$$

(19) $${FeCl}_{2}OH+H^{+}\to {Fe}^{3+}+2{\;Cl}^{-}+H_{2}O$$

(20) $$2{\;PO}_{4}^{3-}+3{\;Ca}^{2+}\to {Ca}_{3}{\left({PO}_{4}\right)}_{2}↓$$

(21) $$2{\;PO}_{4}^{3-}+3{\;Mg}^{2+}\to {Mg}_{3}{\left({PO}_{4}\right)}_{2}↓$$

(22) $$2{\;PO}_{4}^{3-}+3{\;Fe}^{2+}+8{\;H}_{2}O\to {Fe}_{3}{\left({PO}_{4}\right)}_{2}\cdot 8{\;H}_{2}O$$

(23) $${PO}_{4}^{3-}+{Fe}^{3+}\to {FePO}_{4}↓$$

While the typical brownish corrosion deposits (corresponding to the products formed during reactions described in Equations (12)–(15)) were found on the surface of the iron sample (Figure 9a), no signs of iron corrosion were spotted in the case of the Fe-Zn specimen even though the weight ratio of initial powders was 1:1. Degradation products deposited on the surface of Fe-Zn samples corresponded to that found on the surface of pure Zn (Figure 9b). Corrosion deposits with similar morphology were also found in a study of Dong [41] where zinc corrosion was studied in the simulated body fluids (SBF) and were identified as calcium phosphates. Since Hanks´ solution also contains $HCO_{3}^{-}$ and $Ca^{2+}$ ions, precipitation of calcium phosphate took place in the case of Zn and Fe-Zn compressed samples, as can be seen in Figure 9b, c. Similarly, in the study of Liu [43], the same corrosion products were found after degradation in Hanks´ solution. Recently, Shen et al. studied the biodegradability and mechanical integrity of a poly(d,l-lactide)(PDLLA)–Zn-nitrided Fe bioresorbable scaffold [47] where Zn was used as a nanoscale sacrificial layer. Within the first 2 months of implantation in the aortas of New Zealand white rabbits, any brown biodegradation products showed which suggest minimal degradation of the Fe platform. Similar behavior was observed for our Fe-Zn sample, even though the zinc layer was not deposited on the Fe platform as a layer but homogenously distributed in the metallic mixture, which can serve as a starting mixture for the further sintering process. Acceleration of the corrosion rate of pure iron by zinc ion implantation was studied by Huang et al. [48], and the enhanced and more uniform corrosion of Fe was also observed after the Zn addition. Three main reasons were identified for such a behavior: lower corrosion potential of Zn and the higher distortion energy after ion implantation, the lower standard electrode potentials of Fe-Zn solid solutions and the uniform distribution of galvanic cells on the Fe layer. Since none other than pure Fe and Zn phases were identified in our mixed sample, no Fe-Zn solid solutions were present. However, after the sintering process, these may be also formed and change the degradation behavior even more due to the galvanic effects described above.

The electrochemical corrosion potential of pure zinc studied in SBF [41] was slightly higher than −1.0 V, while results for our sample were −1.05 ± 0.1 V. Chen [42] observed the potential of −0.99 V in PBS and similarly Kubásek [49] the potential of −0.98 V. A slight shift to the more negative values observed for our sample is associated to the difference in the sintered and raw sample and its higher corrosion susceptibility due to the higher porosity and different phase structure. Values of *E_corr_* obtained for pure iron (−0.68 ± 0.1), however, differ from the ones that can be found in the literature for sintered iron. Zhang [46] found *E_corr_* of pure Fe to be −0.51 V, but more negative corrosion potentials (from −0.718 to −0.748) were also reported [50,51]. This finding emphasizes the influence of the preparation method on the corrosion properties of biodegradable metals [33]. Electrochemical behavior of the mixed Fe-Zn sample resembled the behavior of pure zinc which can be attributed to the effect of cathodic protection of iron by zinc where zinc acted as a sacrificial anode [52]. In the case of sintered samples, where 1 to 5 wt% of iron was added to the zinc sample, this effect was not observed due to the creation of different Fe-Zn phases which enhanced the corrosion rates of the studied material [25].

## 5. Conclusions

Metallic powders were compressed using a hydraulic press, and their degradation properties in simulated body fluids represented by Hanks´ solution were studied. A 21-day-long immersion test was carried out to characterize corrosion deposits created on the metallic sample surfaces, and electrochemical methods have been chosen for corrosion rate evaluation. Samples from pure Zn, Fe and mixed Fe-Zn (in a weight ratio of 1:1) were prepared and characterized by the SEM, EDX and XRD methods. Homogeneous distribution of Fe and Zn powder particles was found in the Fe-Zn sample. Degradation tests showed that the mixed sample showed no to minimal signs of iron corrosion while the zinc degradation occurred. These results were also supported by the analysis of ions’ concentration in the solution after immersion where no iron was detected for the Zn-Fe sample. Corrosion deposits in the form of calcium phosphates formed on the Zn and Fe-Zn samples, and their electrochemical characteristics were similar as well. This was because zinc acted as a sacrificial anode and protected the iron from degradation. The fastest corrosion rate was observed for pure zinc followed by the Fe-Zn and Fe samples with the corrosion rate of Fe-Zn (0.491 ± 0.04) shifted to the values measured for pure zinc. The characterization of corrosion properties of powders and powder mixes may help to fabricate biodegradable implants and to understand their behavior in the environment of simulated body fluids better. By the changes in the weight ratio of initial powders, corrosion properties can be influenced in an easy and controllable manner.

## Figures and Tables

**Table 1:** Electrochemical parameters of Zn, Fe and Fe-Zn compressed metallic powders measured in Hanks´ solution at 37 ± 2 °C.

Sample	*E_corr_ (V)*	*j_corr_* (µA·cm^−^²)	Corrosion Rate (mm·year^−1^)	Polarization Resistance (Ω)
Zn	−1.05 ± 0.10	37 ± 2.9	0.549 ± 0.07	140 ± 32
Fe	−0.68 ± 0.10	18 ± 4.1	0.209 ± 0.11	339 ± 58
Fe-Zn	−0.97 ± 0.04	43 ± 2.9	0.491 ± 0.04	164 ± 28

**Table 2:** Changes in pH after 7 and 21 days of material immersion in Hanks´ solution at 37 ± 0.2 °C with corresponding ions concentrations determined by atomic absorption spectroscopy (AAS).

Sample	pH after 7 Days	ΔpH after 7 Days	Ions Concentration (mg·L^−1^) after 7 Days		pH after 21 Days	ΔpH after 21 Days	Ions Concentration (mg·L^−1^) after 21 Days	
			Fe	Zn			Fe	Zn
Zn	9.28 ± 0.05	+1.88	-	6.04 ± 0.7	7.63 ± 0.02	+0.23	-	7.42 ± 0.8
Fe	9.55 ± 0.11	+2.15	0.94 ± 0.5	-	7.71 ± 0.07	+0.31	0.26 ± 0.2	-
Fe-Zn	9.58 ± 0.08	+2.18	Non-detectable	6.14 ± 0.6	7.65 ± 0.07	+0.25	Non-detectable	6.04 ± 0.7

## Data Availability

The data presented in this study are available on request from the corresponding author.
